# Verifying the calibration of a manual one-position keratometer

**Published:** 2013

**Authors:** Ismael Cordero

**Affiliations:** Clinical Engineer, Philadelphia, Pennsylvania, United States ismaelcordero@me.com

**Figure:**
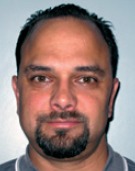
Ismael Cordero

A keratometer, also known as an ophthalmometer, is a diagnostic instrument for measuring the curvature of the anterior surface of the cornea, which is used to assess the amount and axis of astigmatism. A very popular and reliable type of manual, one-position keratometer (commonly known as a Bausch and Lomb-type keratometer) is shown in Figure 1. Although this type of keratometer rarely goes out of calibration, it should be checked for accuracy at least once a year.

## Procedure

To verify the accuracy of the keratometer you need a set of standard spheres, which many manufacturers include with the keratometer. Such a set typically includes three highly polished steel balls of known curvatures (for instance, 40.50, 42.50, and 44.75 diopters) and a magnetised mounting device that attaches to the headrest of the keratometer (Figure 1).

### Adjust the eyepiece

Do not omit this step. If you do not adjust the eyepiece, you may think that your keratometer is out of adjustment when it may not be.

1. Place a sheet of white paper over the back of the keratometer. The white background will better highlight the crosshairs when viewed through the eyepiece.
2. Turn on the instrument.
3. Rotate the eyepiece fully counterclockwise. You will notice that the crosshairs will become blurred, and thus inhibit accommodation.
4. While keeping both eyes open, turn the eyepiece in the clockwise (plus) direction until the crosshairs come into sharp focus, then stop. The keratometer has now been adjusted for your refractive error.

**NOTE:** Do not move the eyepiece back and forth in the plus and minus directions; only approach the point of focus from the plus direction.

### Mount the test sphere

1. Secure the sphere mount to one side of the keratometer's headrest.
2. Place a test sphere (for example, the 42.50 diopter sphere) on the magnetic mount.
3. Rotate the mount towards the keratometer so that the test sphere is where a patient's eye would normally be positioned.

**Figure:**
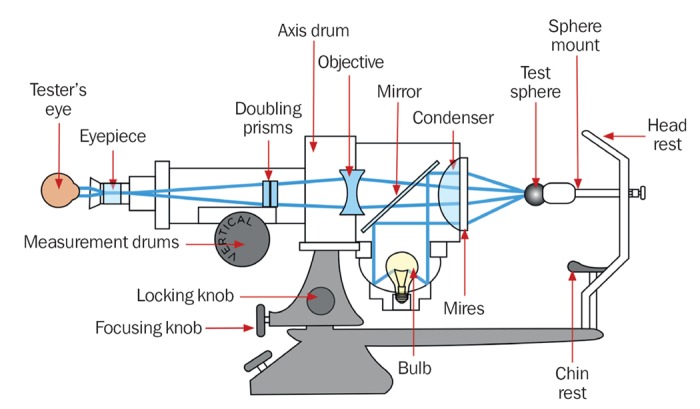
Figure 1

**NOTE:** If the sphere has a dull appearance it may not sufficiently reflect the mires, making measurements difficult. If it is dull, use a soft cotton cloth to polish its surface.

### Focus the mires

1. When looking through the eyepiece, you will observe three circles, each with a plus (+) sign to its left and to its right, and a minus (−) sign on top and below. The bottom right-hand circle should have the crosshair in the centre and may appear doubled (Figure 2a).
2. Using the focusing knob, bring the bottom right circle into focus as a single image (Figure 2b).
3. Lock the instrument into place using the locking knob. This will ensure that the instrument does not rotate during the measurement process.

### Measure the test sphere

**NOTE:** since the test spheres are completely spherical, there is no need to rotate the axis drum on the top of the unit so that the plus (+) signs of the adjacent circles are on the same plane, as would be required when assessing a real eye.

**Figure:**
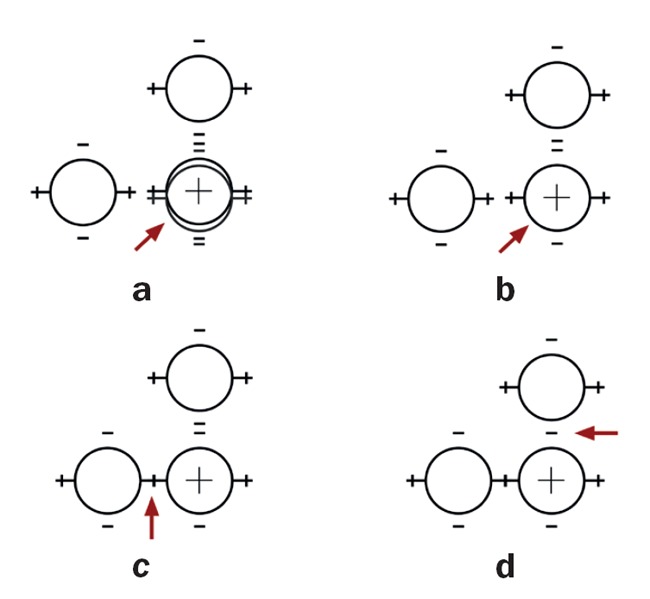
Figure 2

1. Turn the horizontal measuring drum and bring the tips of the plus signs together until they are superimposed (Figure 2c) and note the reading on the horizontal measuring drum (Figure 3a).
2. Turn the vertical measuring drum and bring the minus signs of the circles above one another together until they are superimposed (Figure 2d) and note the reading on the vertical measuring drum (Figure 3b).

If both the readings on the horizontal and vertical measuring drums match the diopter value of the sphere (plus or minus an eighth of a diopter), then the keratometer is accurate. If you have other spheres, you can repeat the procedure to confirm the calibration.

If you find the keratometer to be out of calibration, the instrument should be calibrated by a professional ophthalmic equipment technician.

**Figure:**
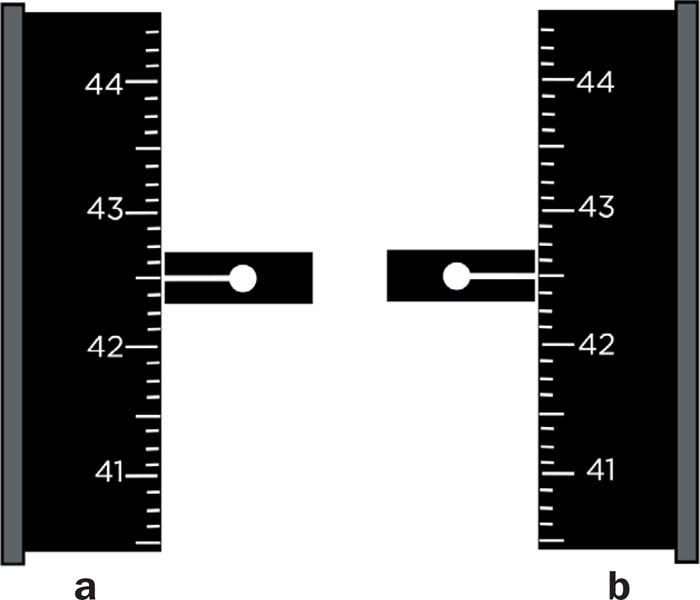
Figure 3

